# The Use and Abuse of the Teeth

**Published:** 1887-06

**Authors:** Lou. P. Bethel


### THE DENTAL REGISTER.

VOL. XLI.] JUNE, 1887. [No. 6.

### COMMUNICATIONS.

### The Use and Abuse of the Teeth.

BY LOU. P. BETHEL, D.D.S.

We truly live in an enlightened age, for never in the history of the world's nations has there been so prosperous a time as now, or as great and as prosperous a country as our own. More grain is raised in this than any other country or nation on the globe, and there is also more consumed, which clearly demonstrates the fact that the American people are high livers. Furthermore it may be said that this fast living is deteriorating and destructive to different organs of the body in many ways. Yet, where is the man who will not sacrifice almost anything to satisfy that very often unnatural appetite?

Lord Lytton paints the picture in perfect harmony with human nature where he alludes to the subject in his Lucile, as follows:

"Oh! hour of all hours, the most blessed upon earth,
Blessed hour of our dinners!
The land of his birth;
The face of his first love; the bills that he owes;
The twaddle of friends and the venom of foes;
The sermon he heard when at church he last went;
The money he borrowed; the money he spent;
All these things, a man I believe may forget,
And not be the worse for forgetting; but yet,
Never, never, oh never! earth's luckiest sinner
Hath unpunished forgotten the hour of his dinner!
Indigestion, that conscience of every bad stomach,
Shall relentlessly gnaw and pursue with some ache

Or some pain; and, trouble remorseless his last ease
As the Furies once troubled the sleep of Orestes.
We may live without poetry, science, and art;
We may live without conscience, and live without heart;
We may live without friends; we may live without books;
But civilized man cannot live without cooks.
We may live without books—what is knowledge but grieving?
We may live without hope—what is hope but deceiving?
We may live without love—what is passion but pining?
But where is the man who can live without dining?"

Man thinks so much of what he eats, yet far less of how he eats it. Where he may be enlightened in one way he is ignorant and careless in another. We say of one "what beautiful teeth he has," and of another, "what homely ones," according as they have been cared for. In some they are 'a thing of beauty and a joy forever,' as the mild but winning pearls so symmetrically arranged in a pin of glittering gold. They are shown with pride and meet the admiration of all.

Others, and entirely too many, seem to think that their only function is to "chaw with," cleanse themselves thereafter and live as best they can. They receive no attention until they ache, and then the forceps is the first resort. Ah! shame for such persons. He that prizeth not his teeth should be made to gum it through life. It has been stated that "one can determine whether persons are neat or slovenly by the condition of their teeth," in which statement there is more truth than poetry.

Is this then the proper use of the teeth; to let them go uncared for; to permit tartar or calculus to cover them, acids to gnaw at will, and foreign substances to collect around and about them; become vitiated and decompose, until not only the vitality of the teeth succumbs to their poisonous effects but the whole system is impregnated and diseased? We say most emphatically, no!

What then are the proper uses of the teeth? First, let no man be afraid to use his teeth in mastication, for it keeps them as well as the parts about them in a normal healthy condition. Yet, he should use them with discretion. How little the great majority of men think of the proper uses of the teeth or of the bad results of improper mastication.

"Indigestion that conscience of every bad stomach
Will relentlessly gnaw and pursue with some ache,
Or some pain, etc."

Do they stop to consider that this great malady is partially, if not wholly, due to the improper use of the teeth in mastication? The jaws have three movements in mastication, namely: Forward and backward, as in biting, from side to side, as in cutting, and downward. In trituration all three movements occur in rapid succession, and with a little time, food will be crushed and properly prepared for deglutition. It is, therefore, better to take too much than not enough time in performing this important function.

People oftentimes contract the habit of using but one side of the arch in mastication. This should be avoided as the teeth on the other side not only become sore through want of use, but also diseased. Further, there are many instances where the excessive use of one side only has deformed the jaw, increased the size of one parotid gland while the other, as a matter of course, from want of stimulation and use, shrunk up or became decreased in size and in consequence deformity resulted. Nature intended that both sides should be equally used, or the teeth would have been differently arranged.

Now, for all mastication is the principal use of the teeth it is by no means the only one, and although more might be said let us consider some of the other uses. From the experience of many we are convinced that the teeth were not intended for "nut crackers," neither is it advisable to use them as many do instead of steel pliers. Biting hard candy should likewise be avoided, and drinking alternately hot and cold liquids, or taking anything into the mouth that will produce much of a thermal change, for such tends to fracture the enamel, as with perhaps one or two exceptions, all bodies expand by heat and contract by cold.

How few people stop to consider as they gaze upon some beautiful face that its symmetry and beauty can be attributed in a measure to the presence of the teeth supporting as they do the lips and cheeks. That to a certain extent they give expression to the face. That they assist greatly in the art of speech and are articles of beauty in themselves when properly treated and cared for. Singularly enough they are sometimes used to assist in the sense of hearing. What a seemingly absurd statement, yet it has been successfully demonstrated that deaf mutes can be made to hear through the intervention of an audiphone between the teeth. The most disgusting use they are subjected to, however, is the grinding up of the filthy tobacco plant—not only a slovenly habit of the person but a disastrous one to the teeth. They are overworked and abraded until the man has the appearance, as expressed by some one, of having "double teeth all around." Notches in or between the teeth are worn by excessively holding clay pipes.

How often people carelessly probe the teeth with pins, needles, gold toothpicks, etc., where only quill or wood should be used in consequence of the injurious effects liable to occur. Biting thread so often indulged in by the ladies is a dangerous practice.

Again, how careless many are in subjecting the teeth to the action of vegetable acids such as malic in apples, citric in lemons, tartaric in grapes, acetic in vinegar, lactic in beets, also generated by the fermentation of milk and sugar in the mouth; all of which act injuriously on tooth substance. After partaking of any of these the teeth should be brushed and the mouth thoroughly rinsed with pure water or an alkaline wash.

It can be justly said that no organs of the body are subjected to so much abuse as the teeth and I think all will agree with the statement. Now, in conclusion, we can but say let each and every one resort to the care of their teeth and avoid the troubles that inevitably result from the non-observance of this important duty.

